# Heterogeneous trajectories of oral frailty and their determinants in older patients receiving dental implants: a longitudinal study

**DOI:** 10.3389/fpubh.2026.1891290

**Published:** 2026-10-05

**Authors:** Ying Zhang, Nan Lu, Mengyao Liang, Qiufeng Zhu

**Affiliations:** 1 Department of Prosthetics, Affiliated Nantong Stomatological Hospital of Nantong University, Nantong, China; 2 Department of Nursing, The Sixth People’s Hospital of Nantong, Nantong, Jiangsu, China; 3 Department of Planting, Nantong Haoting Dental Clinic, Nantong, China

**Keywords:** aged, dental implantation, latent class growth modeling, longitudinal studies, oral frailty

## Abstract

**Objective:**

To describe distinct trajectory patterns of oral frailty after implant-supported prosthesis delivery in older patients and to identify associated factors.

**Methods:**

This prospective longitudinal cohort study consecutively enrolled 211 older patients who completed implant-supported prosthesis delivery at Nantong Stomatological Hospital and Haoting Dental Clinic between January 1 and April 30, 2025. Oral frailty was assessed using the adapted seven-item version of the Oral Frailty Index-8 (OFI-8, 7-item version; score range 0–9) at baseline (T0, prosthesis delivery) and at 3, 6, 9, and 12 months post-delivery (T1–T4). Distinct trajectories were identified using latent class growth modeling (LCGM). Multivariable multinomial logistic regression was used to analyze factors associated with trajectory membership.

**Results:**

Three distinct oral frailty trajectories were identified: (1) Rapid Improvement-Stable Pattern (Class 1, *n* = 90, 42.7%), characterized by a sharp decline in OFI-8 scores immediately after prosthesis delivery followed by sustained low levels; (2) Gradual Improvement-Plateau Pattern (Class 2, *n* = 74, 35.1%), showing a gradual decline followed by a plateau; and (3) Persistent High-Risk Pattern (Class 3, *n* = 47, 22.2%), with scores remaining elevated throughout the 12-month observation period. Multivariable multinomial logistic regression analysis revealed that age (aOR = 1.12, 95% CI: 1.04–1.21), number of medications (aOR = 1.25, 95% CI: 1.05–1.49), and number of missing teeth (aOR = 1.09, 95% CI: 1.02–1.16) were independently associated with membership in Class 3. Bootstrap validation supported the robustness of the model. None of these variables showed statistical significance in distinguishing Class 2 from Class 1.

**Conclusion:**

Oral frailty trajectories in older patients with dental implants are heterogeneous. Older age, polypharmacy, and a greater number of missing teeth were independently associated with membership in the persistent high-risk trajectory, suggesting that these factors may help identify patients requiring prioritized follow-up.

## 1 Introduction

The global aging demographic structure is profoundly reshaping public health priorities. In this context, maintaining the integrity of oral function in older adults has transcended the traditional scope of caries and periodontal disease prevention, emerging as a core concern intricately linked to nutritional status, systemic health, and quality of life (1). Oral frailty, an emerging composite concept, encapsulates the multidimensional decline in oral function associated with aging. Its clinical manifestations encompass decreased masticatory efficiency, impaired swallowing coordination, reduced oral self-cleaning capacity (e.g., xerostomia), and diminished speech clarity (2–4). Epidemiological data reveal that the overall prevalence of oral frailty in community-dwelling older adults can reach 29.5%, with an even higher proportion, 47.9%, in a pre-frail state, highlighting its pervasiveness (5). More critically, oral frailty exhibits significant associations with malnutrition, sarcopenia, cognitive decline, and increased all-cause mortality (6, 7), positioning it as a key, modifiable risk nexus in the process of healthy aging.

Implant-supported prostheses, owing to their favorable long-term survival rates and functional restorative capacity, have become the standard of care for the rehabilitation of partial and complete edentulism (8). The Chinese dental implant market has experienced substantial annual growth, with older patients constituting a primary demographic (9). However, the prevailing paradigm for evaluating therapeutic success remains predominantly focused on local biomechanical and structural parameters, such as peri-implant marginal bone levels, soft tissue aesthetics, and the mechanical integrity of the prosthesis (10, 11). This paradigm harbors a fundamental limitation: it emphasizes the success of the replacement prosthetic unit itself, while inadequately evaluating its long-term impact on the host’s overall oral functional ecosystem. Achieving successful implant osseointegration and loading addresses the anatomic deficiency of a local masticatory unit but may not concurrently reverse or ameliorate the systemic decline in oral function driven by aging, multimorbidity, diminished neuromuscular control, and behavioral factors.

Consequently, a critical scientific question demands an answer: Following the successful delivery of an implant-supported prosthesis, what longitudinal trajectory will the overall oral frailty status of older patients follow? Existing research primarily employs cross-sectional designs or short-term pre-post comparisons, which fail to unveil the temporal patterns and population heterogeneity inherent in this dynamic process (12, 13). Given the substantial heterogeneity among older patients in baseline nutritional status, comorbidity burden, and oral foundational status, their trajectories of functional adaptation and benefit post-rehabilitation are likely to vary significantly.

Therefore, this study employed a prospective longitudinal cohort design to describe the dynamic trajectories of oral frailty following definitive implant-supported prosthesis delivery in older adult patients. The specific objectives were: (1) to identify and quantify distinct trajectory subgroups of oral frailty over the 12-month period after prosthesis delivery using latent class growth modeling; and (2) to analyze the associations of demographic, oral health, and systemic health factors with trajectory membership. From a heterogeneity perspective, this study contributes to a deeper understanding of long-term functional outcomes after implant rehabilitation and provides evidence to support patient-centered functional outcome monitoring and individualized management in clinical practice.

## 2 Subjects and methods

### 2.1 Study subjects

This study was designed as a prospective longitudinal cohort study. Participants were consecutively enrolled from among older patients who underwent the delivery of the definitive implant-supported prosthesis at the Department of Implant Prosthodontics, Nantong Stomatological Hospital, and Haoting Dental Clinic, Nantong, between January 1, 2025, and April 30, 2025.

Inclusion criteria were: (1) Age ≥60 years; (2) Scheduled to receive an implant-supported fixed prosthesis for partial or complete edentulism; (3) Having successfully undergone the delivery of the definitive prosthesis without acute discomfort; (4) Being conscious and possessing basic communication and comprehension abilities, capable of completing questionnaires and interviews independently or with minimal assistance; (5) Voluntary participation with written informed consent provided. Exclusion criteria were: (1) Presence of severe, uncontrolled systemic diseases with an expected survival or health status incompatible with completion of the 1-year follow-up; (2) Having undergone complex concomitant jawbone reconstruction procedures (e.g., external sinus lift, extensive onlay bone grafting) simultaneous with implant placement; (3) Presence of untreated active periodontitis or active peri-implant infection.

Systemic health status assessment: medical history, including common chronic conditions such as hypertension, diabetes mellitus, coronary heart disease, and stroke, was collected through the electronic medical record system (diagnoses were confirmed based on specialist documentation from tertiary hospitals or long-term medication history). Current medication lists, including the types and dosages of both prescription and over-the-counter drugs, were recorded, and the total number of medication types was calculated. Smoking history was obtained through self-report and categorized as current/former smoking or never smoking.

For latent class growth modeling, sample size requirements depend on factors such as the number of classes, degree of separation between classes, number of indicators, and effect size. Monte Carlo simulation studies have suggested that when class separation is adequate (entropy >0.8) and the number of classes is three, a total sample size of at least 200 participants, with each class containing at least 50 participants, is recommended to ensure unbiased parameter estimates and accurate standard errors (14). A total of 232 patients were enrolled at baseline. Over the 12-month follow-up period, 21 patients were excluded due to voluntary withdrawal, loss to contact, or development of other severe illnesses that prevented completion of all five assessments, yielding an overall attrition rate of 9.1%. Ultimately, 211 patients completed all five time-point assessments and were included in the final statistical analysis. This study was approved by the Ethics Committee of Nantong Stomatological Hospital (Approval No.: PJ2025-11-01), and all participants provided written informed consent.

### 2.2 Study instruments

#### 2.2.1 General Information Questionnaire

Designed by the research team, this questionnaire collected the following data: patient age, gender, years of education, living arrangement, primary caregiver, presence of chronic diseases, number of medications taken, self-reported health status, nutritional status assessed using the Mini Nutritional Assessment Short-Form (MNA-SF) (15), number of remaining natural teeth, status of the opposing dentition, periodontal health status, number of implants placed, implant location(s), type of prosthesis, and whether bone augmentation procedures were performed.

#### 2.2.2 Oral frailty index-8 (OFI-8)

Oral frailty was assessed using the Chinese version of the OFI-8, which has been validated for reliability and validity (16). The scale consists of 8 items covering five dimensions: chewing ability, swallowing function, use of dentures, social participation, and oral health-related behaviors. Items 1–3 (difficulty chewing, choking while swallowing, use of dentures) are scored from 0 to 2, and items 4–8 (dry mouth, reduced going-out frequency, difficulty chewing hard foods, tooth brushing frequency, and dental visit frequency) are scored from 0 to 1. The total score ranges from 0 to 11, with higher scores indicating greater risk of oral frailty. Given that all participants in this study received implant-supported fixed prostheses, the original item “use of dentures” (item 3) was no longer applicable. After discussion within the research team, this item was removed, and the remaining seven core items were retained. Among them, the two items regarding difficulty chewing and choking while swallowing were each scored from 0 to 2, and the five items regarding dry mouth, reduced going-out frequency, difficulty chewing hard foods, tooth brushing frequency, and regular dental visits were each scored from 0 to 1. The adjusted total score ranges from 0 to 9, with higher scores indicating greater risk of oral frailty. The Cronbach’s α coefficient of the adapted scale was 0.93, indicating good reliability in this population of implant-supported prosthesis patients. Validation studies of this scale in Chinese older adults have demonstrated satisfactory validity, with total scores significantly correlated with physical function, nutritional status, and other indicators (17).

### 2.3 Data collection and quality control

This study employed an observational longitudinal design. The date of definitive prosthesis delivery was defined as the study baseline (T0), with prospective, multi-timepoint follow-ups conducted thereafter. Pre-specified follow-up time points were: T1 (3 months ± 2 weeks post-delivery), T2 (6 months ± 2 weeks), T3 (9 months ± 2 weeks), and T4 (12 months ± 2 weeks). All follow-up assessments were performed during scheduled patient recall visits by the same, specifically trained research nurse to ensure consistency in evaluation.

To ensure data integrity and accuracy, a unified electronic Case Report Form (e-CRF) was used for on-site data collection and immediate verification. Subsequently, two independent data administrators performed dual data entry, followed by comparison and logical consistency checks. Follow-up timeliness was managed by adhering to a predefined time window (±2 weeks) and employing a dedicated study coordinator to schedule patient appointments and send reminders.

Further quality control measures were implemented. Midway through the study, a random subset of cases underwent blinded duplicate assessment to monitor inter-rater reliability. Furthermore, the assessor was kept blinded to the patients’ eventual trajectory group classification. These combined measures aimed to minimize various biases and ensure a high-quality data foundation for subsequent analyses.

### 2.4 Statistical analysis

Data analysis was conducted using R software (version 4.3.0) and the “lcmm” package. Normality of continuous variables was assessed using the Kolmogorov–Smirnov test. Continuous variables conforming to a normal distribution are presented as mean ± standard deviation (x̄ ± s). Categorical variables are presented as frequency (percentage) [*n* (%)].

First, linear and nonlinear unconditional latent growth curve models were fitted to explore the overall trend of oral frailty scores over time after prosthesis delivery. Subsequently, latent class growth modeling (LCGM) was performed to identify distinct trajectory subgroups. Models with 1 to 5 latent classes were sequentially fitted, and the optimal number of classes was determined by comparing fit indices, including the Akaike Information Criterion (AIC), Bayesian Information Criterion (BIC), sample-size adjusted BIC (aBIC), entropy (values >0.8 indicating good classification), the Lo–Mendell–Rubin likelihood ratio test (LMR-LRT), and the bootstrap-based likelihood ratio test (BLRT). The most parsimonious model with the best fit was selected based on a comprehensive evaluation of these indices and model interpretability. Each trajectory subgroup was named and described according to its pattern of oral frailty scores across the five time points (e.g., initial level, slope, and shape of change). After determining the optimal trajectory model, differences in baseline sociodemographic characteristics, systemic health status, and oral and implant-related clinical features across trajectory subgroups were compared using one-way ANOVA, Kruskal-Wallis *H* test, or chi-square test, as appropriate. Using trajectory subgroup membership as the dependent variable, variables with *p* < 0.10 in univariate analysis were entered as candidate independent variables into a multinomial logistic regression model to identify factors independently associated with trajectory membership. Adjusted odds ratios (aOR) and their 95% confidence intervals (CI) were calculated. To evaluate the robustness of the findings, three sensitivity analyses were conducted. First, a bootstrap internal validation (1,000 resamples) was performed for the multivariable logistic regression model, comparing effect estimates and confidence intervals between bootstrap samples and the original sample to assess model stability. Second, OFI-8 scores were treated as count variables and re-fitted using a Poisson-distributed LCGM with robust standard errors to account for potential overdispersion, thereby testing the sensitivity of trajectory classification to distributional assumptions. Third, OFI-8 scores were categorized into tertiles (low: 0–3; medium: 4–6; high: 7–9) and re-fitted using a multinomial-distributed growth model to test the sensitivity of trajectory classification to variable scaling. Consistency between the trajectory classifications from these sensitivity analyses and the primary analysis was assessed using the kappa coefficient. To examine the longitudinal measurement invariance of the OFI-8 (7-item version), longitudinal confirmatory factor analysis (LG-CFA) was conducted. Four nested models were fitted sequentially: configural (M1), weak (M2), strong (M3), and strict (M4) invariance, constraining the factor structure, factor loadings, item thresholds, and residual variances across time, respectively. Models were estimated using the robust weighted least squares estimator (WLSMV), and residual correlations of the same item across adjacent time points were allowed. Model comparisons were based on ΔCFI ≤0.01 and ΔRMSEA ≤0.015. Analyses were performed using Mplus 8.0.

During data collection, no item-level missing data for OFI-8 were observed among patients who completed all follow-up assessments. For missing data due to attrition, a complete-case analysis was adopted, including only patients who completed all five follow-up assessments (*n* = 211) in the final analysis. To evaluate potential attrition bias, baseline demographic characteristics, oral health status, and systemic health indicators were compared between completers and dropouts. The latent class growth model entails the following assumptions: (1) observed indicators at each time point follow a normal distribution within each class; (2) between-individual variability within each class is captured by random effects (random intercepts and random slopes), with residuals independently and identically distributed; and (3) class membership is conditionally independent given the latent variable. To evaluate these assumptions, the following diagnostic checks were performed. First, after fitting the 3-class model, standardized residuals at each time point were extracted and tested for normality. The standardized residuals exhibited skewness ranging from −0.15 to 0.22 and kurtosis ranging from −0.31 to 0.45, with Shapiro–Wilk test *p*-values all exceeding 0.05, supporting approximate normality of the residuals. Second, class separation was evaluated using entropy (0.892) and average posterior probabilities (all >0.90), with high classification quality indicating that the within-class homogeneity assumption was largely satisfied. Third, the conditional independence assumption was assessed by comparing model fit under different residual covariance structures (equal residuals vs. freely estimated residuals). The more parsimonious model with equal residuals yielded a lower BIC value, supporting this assumption.

## 3 Results

### 3.1 Patient baseline characteristics and follow-up

A total of 232 patients were enrolled at baseline. The patient enrollment, follow-up, and attrition process is illustrated in Figure 1. In addition to the baseline assessment on the day of definitive prosthesis delivery (T0), all participants were scheduled for reassessment at 3, 6, 9, and 12 months post-delivery (T1–T4). Over the 12-month follow-up period, 21 patients failed to complete all five assessments due to voluntary withdrawal, loss to contact, or development of other severe illnesses, yielding an overall attrition rate of 9.1%. Specifically, 4 patients were lost at the 3-month follow-up (2 withdrew, 2 were unreachable); the cumulative number of dropouts reached 9 at the 6-month follow-up (3 new withdrawals, 2 new cases of other illnesses); 15 at the 9-month follow-up (2 new withdrawals, 3 newly unreachable, 1 new case of other illness); and 21 at the 12-month follow-up (3 new withdrawals, 2 newly unreachable, 1 new case of other illness). Ultimately, 211 patients (90.9%) completed all follow-up assessments and were included in the final analysis. To evaluate potential attrition bias, baseline characteristics were compared between completers (*n* = 211) and dropouts (*n* = 21), as shown in Table 1. No statistically significant differences were observed between the two groups in terms of age, sex, number of missing teeth, number of medications, chronic disease history, or baseline OFI-8 (7-item version) scores (all *p* > 0.05), suggesting that the impact of attrition on the study findings may be limited.

**Figure 1:**
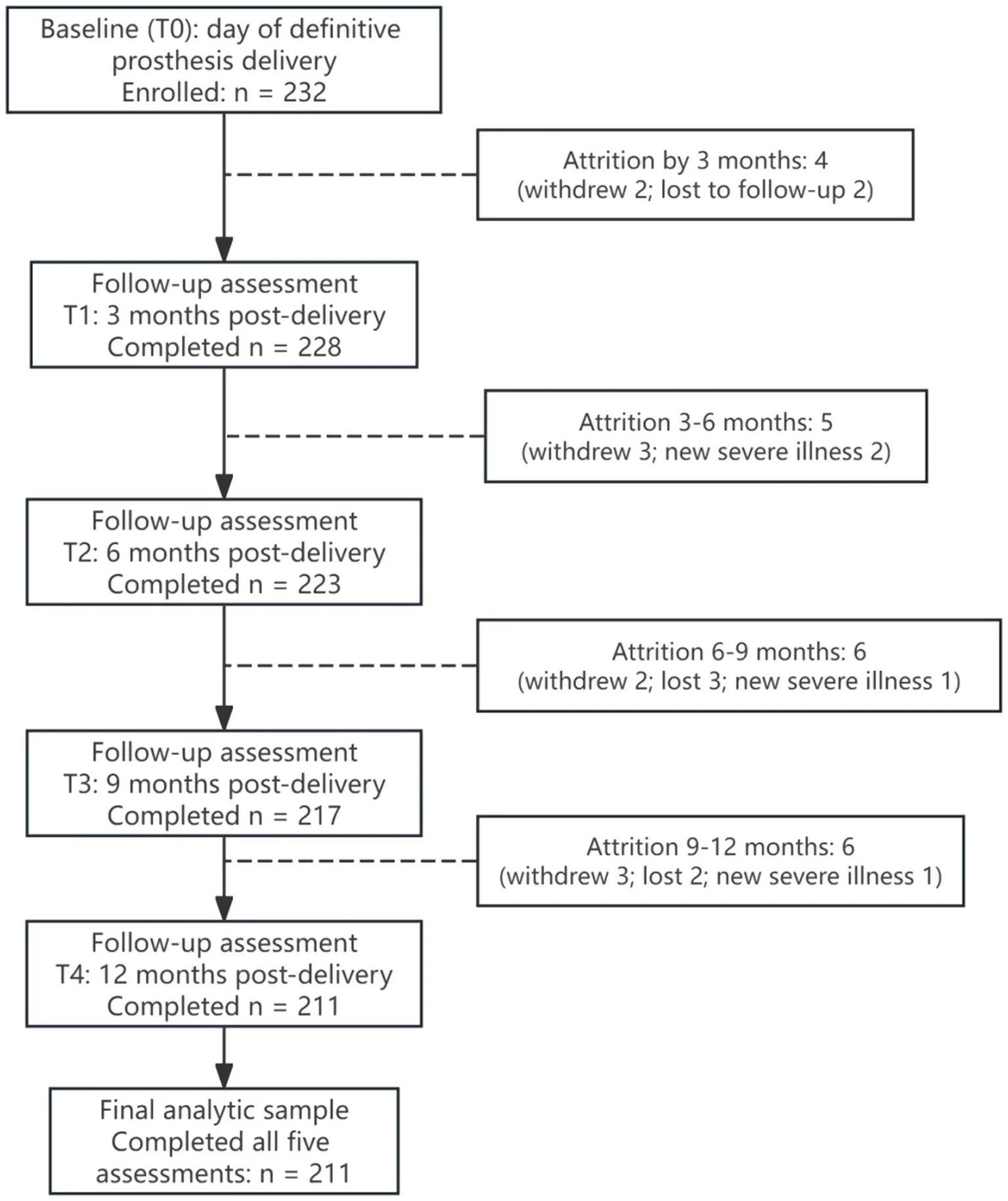
Flow diagram of patient enrollment, follow-up, and attrition. T0 indicates the day of definitive prosthesis delivery (baseline). T1-T4 represent follow-up assessments at 3, 6, 9, and 12 months post-delivery.

**Table 1:** Baseline characteristics of the study participants (*n* = 211).

Variable	Category/statistic	*n* (%)/descriptive statistic
Sociodemographic characteristics		
Age (years)		68.4 ± 5.6
Gender	Male	98
	Female	113
Education level	Junior high school or below	87
	High school/Technical secondary school	76
	College/University or above	48
Living arrangement	Living alone	31
	Living with family	172
	Institutional care	8
Systemic health status		
Number of chronic diseases	0–2	145
	≥3	66
Number of medications (types)		2.8 ± 2.0
Mini-nutritional assessment (MNA-SF) score	Normal nutrition	162
	At risk of malnutrition	46
	Malnourished	3
Smoking history	Current or former smoker	85
	Never smoked	126
Oral and implant-related characteristics		
Number of remaining natural teeth		211
Status of opposing dentition	Natural dentition/Implant prosthesis	156
	Removable denture	55
Type of current prosthesis	Single crown	124
	Splinted crown or fixed dental prosthesis	87
Number of implants placed	1–2	178
	≥3	33
Bone augmentation surgery	Yes	67
	No	144

### 3.2 Measurement invariance testing

To examine whether the OFI-8 (7-item version) measured the same construct across different time points, longitudinal confirmatory factor analysis was conducted to evaluate the measurement invariance of the scale. The results showed that the configural invariance model (M1) fit the data well (CFI = 0.965, RMSEA = 0.048). Compared with M1, the weak invariance model (M2, constraining factor loadings to be equal across time) did not show significant deterioration in fit (ΔCFI = 0.004, ΔRMSEA = 0.001), supporting weak invariance. The strong invariance model (M3, additionally constraining item intercepts to be equal across time) also demonstrated acceptable fit (ΔCFI = 0.008, ΔRMSEA = 0.004). However, the strict invariance model (M4, constraining residual variances to be equal across time) yielded a ΔCFI of 0.012, which exceeded the recommended threshold, and was therefore not supported. These findings indicate that the OFI-8 (7-item version) had satisfactory weak and strong invariance across different postoperative rehabilitation stages, with the meanings and thresholds of the items being generally consistent across time points. Thus, the identified trajectories reflect genuine changes in oral frailty rather than changes in the measurement properties of the instrument (see Table 2).

**Table 2:** Measurement invariance test results of the oral frailty scale across time points.

Model	χ^2^	df	CFI	RMSEA (90% CI)	ΔCFI	ΔRMSEA
Configural invariance (M1)	185.42	112	0.965	0.048 (0.039–0.057)	—	—
Metric invariance (M2, equal factor loadings)	198.76	118	0.961	0.049 (0.040–0.058)	0.004	0.001
Scalar invariance (M3, equal item intercepts)	214.35	125	0.953	0.053 (0.044–0.062)	0.008	0.004
Strict invariance (M4, equal error variances)	243.18	132	0.941	0.061 (0.052–0.070)	0.012	0.008

CFI, comparative fit index; RMSEA, root mean square error of approximation; ΔCFI and ΔRMSEA represent changes relative to the preceding model. According to Chen (30), ΔCFI ≤ 0.01 and ΔRMSEA ≤ 0.015 indicate support for measurement invariance.

### 3.3 Overall trend of oral frailty scores

Descriptive statistics of the OFI-8 total scores for all patients over the 12-month observation period are presented in Table 3 and Figure 2. At baseline (T0, the day of prosthesis delivery), the mean OFI-8 score was 5.3 ± 2.1. Following delivery, scores showed a significant declining trend. By 3 months post-delivery (T1), the mean score had decreased to 3.8 ± 1.9. The scores reached their lowest point during the observation period at 6 months post-delivery (T2), with a mean of 3.2 ± 1.7. In subsequent follow-ups, scores showed a slight increase but remained significantly lower than baseline levels, with mean scores of 3.5 ± 1.8 and 3.6 ± 1.9 at 9 months (T3) and 12 months (T4) post-delivery, respectively. Repeated measures analysis of variance (RM-ANOVA) revealed a statistically significant difference in OFI-8 total scores across the different time points (*F* = 45.72, *p* < 0.001). Post-hoc pairwise comparisons indicated that scores at all post-delivery time points (T1–T4) were significantly lower than the score on the delivery day (T0) (all *p* < 0.001).

**Table 3:** Changes in oral frailty (OFI-8) scores at different time points following prosthesis delivery (*n* = 211).

Time point	Sample size (*n*)	OFI-8 total score (Mean ± SD)	*p*-value vs. baseline (T0)^1^
T0 (delivery day)	211	5.3 ± 2.1	—
T1 (3 months post-delivery)	211	3.8 ± 1.9	<0.001
T2 (6 months post-delivery)	211	3.2 ± 1.7	<0.001
T3 (9 months post-delivery)	211	3.5 ± 1.8	<0.001
T4 (12 months post-delivery)	211	3.6 ± 1.9	<0.001
Overall comparison statistic		*F* = 45.72	*p* < 0.001

^1^Overall comparison was performed using repeated measures ANOVA, with pairwise comparisons adjusted using the Bonferroni method. Scores at T1, T2, T3, and T4 were all significantly lower than at T0 (all *p* < 0.001). The score at T2 was significantly lower than at T1 (*p* = 0.003). There were no statistically significant differences in scores between T3/T4 and T2 (*p* > 0.05), nor between T3/T4 and T1 (*p* > 0.05).

**Figure 2:**
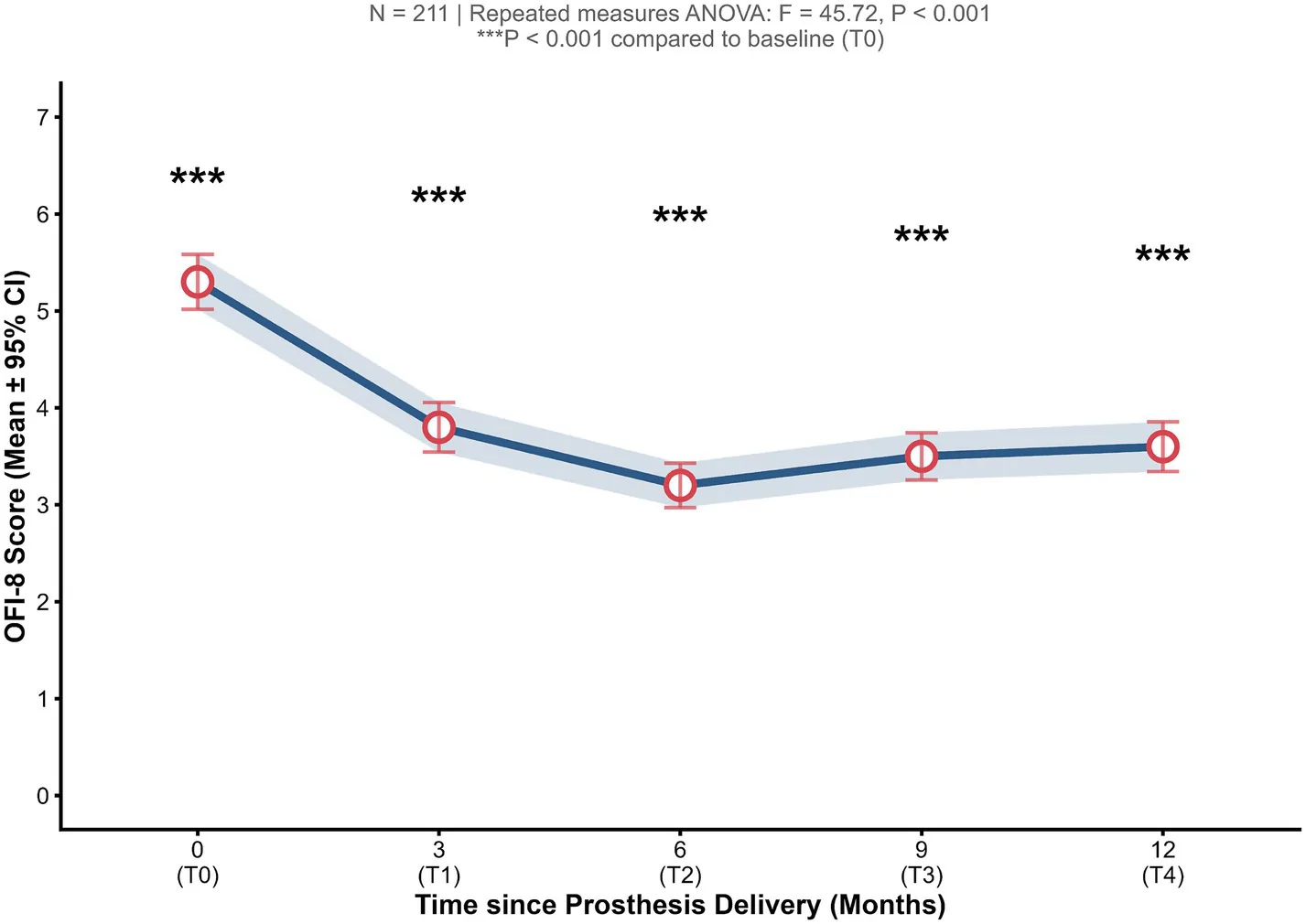
Overall Trajectory of Oral Frailty.

### 3.4 Latent class growth analysis of oral frailty trajectories

#### 3.4.1 Overall trend and determination of optimal class number

To investigate the fundamental trend of changes in the total oral frailty score over time, unconditional latent growth models, both linear and non-linear (quadratic), were initially constructed. The comparison of model fit indices is presented in Table 4. The non-linear (quadratic) model demonstrated significantly better fit across all indicators compared to the linear model: its Comparative Fit Index (CFI) and Tucker-Lewis Index (TLI) were both >0.95, Root Mean Square Error of Approximation (RMSEA) was <0.06, Standardized Root Mean Square Residual (SRMR) was <0.05, and it’s AIC and BIC values were lower. This indicates that the change in the total oral frailty score over the 12 months post-delivery was not a simple linear decline, but rather a curve characterized by an initial rapid decline followed by a leveling-off. Therefore, the subsequent LCGM was constructed based on the non-linear (quadratic) form.

**Table 4:** Comparison of goodness-of-fit indices for unconditional linear and non-linear latent growth models.

Model Type	χ^2^/df	CFI	TLI	RMSEA (90% CI)	SRMR	AIC	BIC
Linear model	8.75	0.891	0.902	0.112 (0.101–0.123)	0.098	4158.33	4189.15
Non-linear (Quadratic) model	4.12	0.968	0.972	0.051 (0.042–0.060)	0.042	4012.47	4049.85

To reveal the heterogeneous patterns of oral frailty changes, latent class growth modeling was applied to analyze OFI-8 scores across five time points in 211 patients. Models with 1 to 5 latent classes were sequentially fitted, and fit indices were compared to determine the optimal number of classes, as shown in Table 5. The AIC values decreased progressively with increasing numbers of classes (1-class: 4210.35; 2-class: 3987.22; 3-class: 3865.48; 4-class: 3859.33; 5-class: 3855.71); however, the rate of decrease markedly slowed after three classes (ΔAIC₃_−_₂ = 121.74, ΔAIC₄_−_₃ = 6.15, ΔAIC₅_−_₄ = 3.62). The BIC reached its lowest value in the 3-class model (3916.04) and increased slightly in the 4- and 5-class models (3922.54 and 3931.57, respectively), indicating that the 3-class model achieved the optimal balance between model fit and parsimony. Furthermore, the 3-class model yielded the highest entropy (0.892), and both the LMR-LRT (*p* = 0.012) and BLRT (*p* < 0.001) were statistically significant, whereas the LMR-LRT *p*-values for the 4- and 5-class models were non-significant (*p* > 0.05), suggesting that adding more classes did not significantly improve model fit. Considering the BIC, entropy, LMR-LRT significance, and model interpretability, the 3-class model was selected as the optimal solution. Further classification diagnostics (Table 5) revealed that the average posterior probabilities for the three classes were 0.93, 0.90, and 0.95, all exceeding the recommended threshold of 0.8. The odds of correct classification (OCC) were 7.8, 5.6, and 9.2, respectively, all greater than 5, indicating excellent classification accuracy. Based on the maximum-probability assignment rule, the actual numbers of patients assigned to each class were: Class 1 (Rapid Improvement-Stable), 90 patients (42.7%); Class 2 (Gradual Improvement-Plateau), 74 patients (35.1%); and Class 3 (Persistent High-Risk), 47 patients (22.2%).

**Table 5:** Comparison of goodness-of-fit indices for latent class growth models with different numbers of classes.

Model (No. of Classes)	AIC	BIC	aBIC	Entropy	LMR-LRT *p* value	BLRT *p*-value	Class probabilities (%)	Average posterior probabilities	OCC
1-Class	4210.35	4235.61	4218.72	—	—	—	100	—	—
2-Class	3987.22	4025.13	3999.41	0.876	<0.001	<0.001	68.2/31.8	0.94/0.91	8.2/6.5
3-Class	3865.48	3916.04	3881.49	0.892	0.012	<0.001	42.7/35.1/22.2	0.93/0.90/0.95	7.8/5.6/9.2
4-Class	3859.33	3922.54	3879.16	0.881	0.124	0.058	40.3/28.4/19.9/11.4	0.89/0.84/0.82/0.79	6.1/4.2/3.8/3.1
5-Class	3855.71	3931.57	3879.36	0.885	0.203	0.105	38.4/27.0/16.1/10.9/7.6	0.83/0.81/0.75/0.73/0.64	5.2/3.8/2.9/2.1/1.8

AIC, Akaike Information Criterion; BIC, Bayesian Information Criterion; aBIC, sample-size adjusted BIC; LMR-LRT, Lo–Mendell–Rubin Likelihood Ratio Test; BLRT, Bootstrap Likelihood Ratio Test; OCC, Odds of Correct Classification.

#### 3.4.2 Clinical characteristics of the three oral frailty trajectories

Based on the results of the 3-class model, the changes in patients’ oral frailty can be clearly categorized into three distinct trajectories, with specific clinical characteristics as follows:

“Rapid Improvement-Stable Pattern”: This class comprised 90 patients (42.7%). Patients in this class had a relatively high baseline OFI-8 score (intercept = 5.92, *p* < 0.001), showing a rapid and significant linear decline within 6 months post-delivery (linear slope = −1.15, *p* < 0.001), followed by a stabilization of the improvement trend (quadratic slope = 0.08, *p* = 0.042). This indicates that this group of patients achieved significant improvement in oral function early after prosthesis delivery, which was maintained during subsequent follow-up.

“Gradual Improvement-Plateau Pattern”: This class included 74 patients (35.1%). Patients in this class had a moderate baseline score (intercept = 4.05, *p* < 0.001), demonstrating a slow but sustained linear improvement after delivery (linear slope = −0.41, *p* = 0.003), with a non-significant quadratic slope (quadratic slope = 0.02, *p* = 0.215), indicating that they reached a plateau in improvement around 9 months post-delivery. Although this group showed improvement, the pace was slower, potentially requiring a longer adaptation period.

“Persistent High-Risk Pattern”: This class consisted of 47 patients (22.2%). Patients in this class had the highest baseline score (intercept = 6.88, *p* < 0.001), but neither the linear nor quadratic slopes were significant over the entire 12-month follow-up period (linear slope = −0.08, *p* = 0.645; quadratic slope = −0.01, *p* = 0.872). This suggests that the implant intervention had limited effect on improving the oral frailty status of this group, and they remained at persistently high risk for oral frailty throughout the observation period. Details are presented in Table 6 and Figure 3.

**Table 6:** Parameter estimates of the 3-class latent class growth model.

Trajectory Class (Designation)	Class probability (*n*, %)	Intercept (initial level)	Linear slope	Quadratic slope	Description of trajectory morphology
Class 1: Rapid Improvement-Stable	90 (42.7%)	5.92 (*p* < 0.001)	−1.15 (P < 0.001)	0.08 (*p* = 0.042)	High baseline level, rapid improvement within 6 months post-delivery, followed by stabilization.
Class 2: Gradual Improvement-Plateau	74 (35.1%)	4.05 (*p* < 0.001)	−0.41 (*p* = 0.003)	0.02 (*p* = 0.215)	Moderate baseline level, slow rate of improvement, plateau reached after 9 months.
Class 3: Persistent High-Risk	47 (22.2%)	6.88 (P < 0.001)	−0.08 (*p* = 0.645)	−0.01 (*p* = 0.872)	Highest baseline level, minimal improvement throughout the 12-month follow-up, persistently high-risk status.

**Figure 3:**
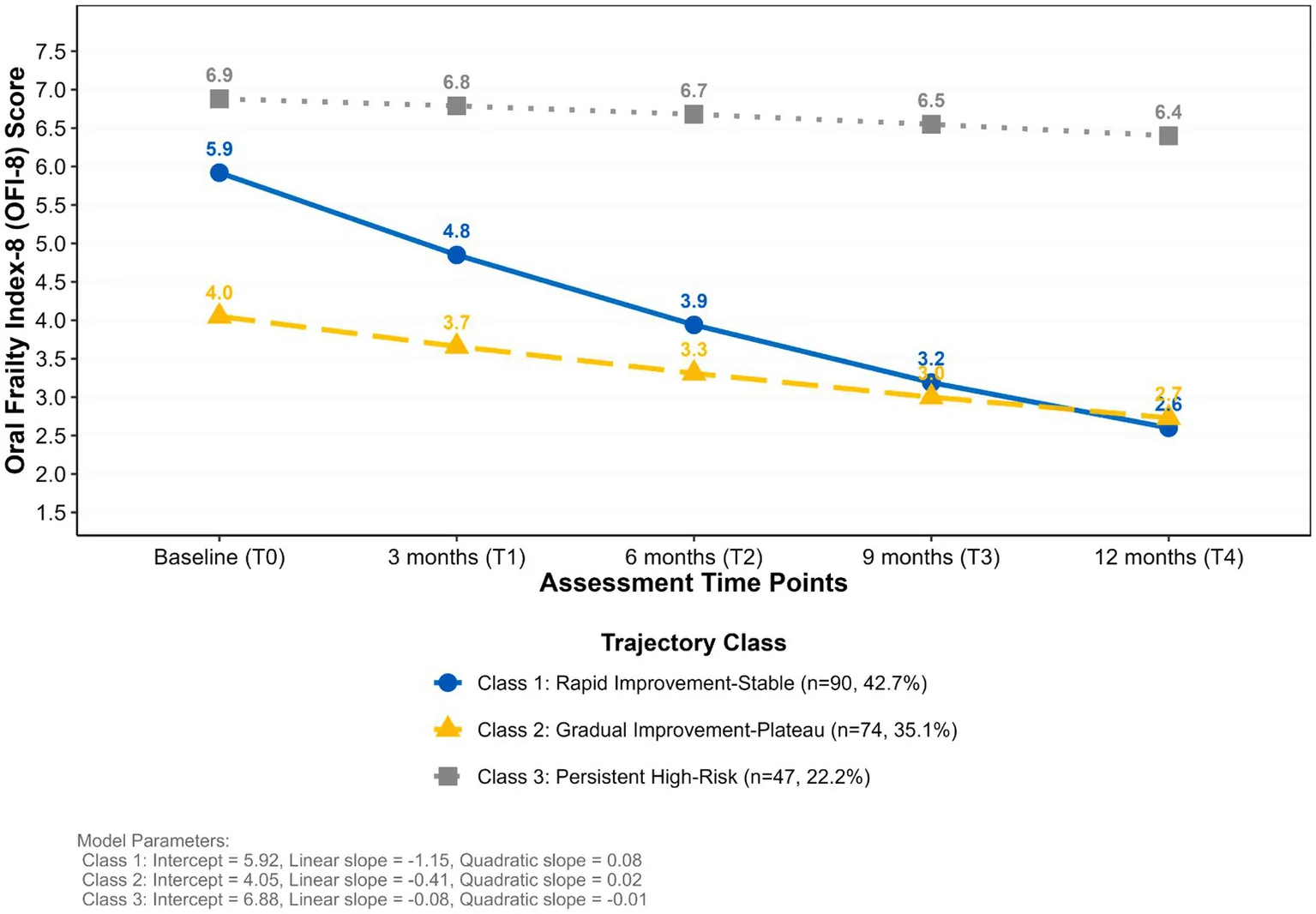
Latent class growth trajectories of oral frailty after implant-supported prosthesis delivery. Three distinct classes were identified: Class 1 (Rapid Improvement), Class 2 (Gradual Improvement), and Class 3 (Persistent High-Risk). Detailed parameter estimates and morphology descriptions are provided in Table 6.

Three distinct classes were identified: Class 1 (Rapid Improvement), Class 2 (Gradual Improvement), and Class 3 (Persistent High-Risk). Detailed parameter estimates and morphology descriptions are provided in Table 6.

### 3.5 Univariate analysis of baseline characteristics among the three oral frailty trajectory groups

To compare differences in baseline characteristics among the different oral frailty trajectory classes, univariate analyses were conducted on various sociodemographic, systemic health, and oral/implant-related variables. The results are presented in Table 7. Significant differences (*p* < 0.05) were found for age, number of medications, and number of remaining natural teeth in at least one between-group comparison.

**Table 7:** Univariate analysis of baseline characteristics among the three oral frailty trajectory groups.

Variable	Category/statistic	Class 1 (*n* = 90)	Class 2 (*n* = 74)	Class 3 (*n* = 47)	Test statistic	*p*-value	Post-hoc comparison
Sociodemographic characteristics							
Age (years)	Mean ± SD	67.8 ± 5.1	69.2 ± 5.4	71.5 ± 5.8	*F* = 5.842	0.003	3 > 1*
Gender	Male	42 (46.7%)	36 (48.6%)	20 (42.6%)	χ^2^ = 0.456	0.796	—
	Female	48 (53.3%)	38 (51.4%)	27 (57.4%)			
Education Level	Junior high or below	39 (43.3%)	34 (45.9%)	22 (46.8%)	χ^2^ = 1.024	0.599	—
	High school/Technical	32 (35.6%)	28 (37.8%)	16 (34.0%)			
	College/University or above	19 (21.1%)	12 (16.2%)	9 (19.1%)			
Living Arrangement	Living alone	14 (15.6%)	11 (14.9%)	6 (12.8%)	χ^2^ = 0.231	0.891	—
	Living with family	73 (81.1%)	61 (82.4%)	38 (80.9%)			
	Institutional care	3 (3.3%)	2 (2.7%)	3 (6.4%)			
Systemic health status							
No. of Chronic Diseases	0–2	64 (71.1%)	49 (66.2%)	32 (68.1%)	χ^2^ = 0.389	0.823	—
	≥3	26 (28.9%)	25 (33.8%)	15 (31.9%)			
No. of Medications (types)	Mean ± SD	2.5 ± 1.8	2.9 ± 2.1	3.4 ± 2.3	H = 6.971	0.031	3 > 1*
MNA-SF Score	Normal nutrition	73 (81.1%)	55 (74.3%)	34 (72.3%)	χ^2^ = 1.876	0.392	—
	At risk of malnutrition	15 (16.7%)	17 (23.0%)	14 (29.8%)			
	Malnourished	2 (2.2%)	2 (2.7%)	1 (2.1%)			
Smoking History	Current/Former smoker	33 (36.7%)	32 (43.2%)	20 (42.6%)	χ^2^ = 0.954	0.621	—
	Never smoked	57 (63.3%)	42 (56.8%)	27 (57.4%)			
Oral and implant-related characteristics							
No. of Remaining Natural Teeth	Mean ± SD	19.5 ± 6.2	17.1 ± 6.5	14.8 ± 7.1	*F* = 9.843	<0.001	1 > 2 > 3**
Status of Opposing Dentition	Natural/Implant	71 (78.9%)	53 (71.6%)	32 (68.1%)	χ^2^ = 2.134	0.545	—
	Removable denture	19 (21.1%)	21 (28.4%)	15 (31.9%)			
Type of Current Prosthesis	Single crown	55 (61.1%)	42 (56.8%)	27 (57.4%)	χ^2^ = 0.231	0.891	-
	Splinted/Fixed bridge	35 (38.9%)	32 (43.2%)	20 (42.6%)			
No. of Implants Placed	1–2	78 (86.7%)	62 (83.8%)	38 (80.9%)	χ^2^ = 0.673	0.713	—
	≥3	12 (13.3%)	12 (16.2%)	9 (19.1%)			
Bone Augmentation Surgery	Yes	26 (28.9%)	24 (32.4%)	17 (36.2%)	χ^2^ = 1.245	0.537	—
	No	64 (71.1%)	50 (67.6%)	30 (63.8%)			

Data are presented as mean ± standard deviation or *n* (%). In the “Test Statistic” column, *F* is from ANOVA, *H* is from the Kruskal-Wallis *H* test, and χ^2^ is from the Chi-square or Fisher’s exact test. Post-hoc comparison: *indicates a statistically significant difference compared to Class 1 (*p* < 0.05); **Indicates statistically significant differences in all pairwise comparisons among the three groups (*p* < 0.05).

### 3.6 Multivariable logistic regression analysis

To investigate factors independently associated with membership in different oral frailty trajectory classes, variables with *p* < 0.1 in the univariate analysis were entered into a multivariable multinomial logistic regression model, with the three-category trajectory as the unordered dependent variable and Class 1 (Rapid Improvement-Stable Pattern) as the reference category.

Class 2 (Gradual Improvement-Plateau Pattern) vs. Class 1 (Rapid Improvement-Stable Pattern): After adjusting for age, number of medications, and number of missing teeth, none of these variables were independently associated with membership in Class 2 (all *p* > 0.05).

Class 3 (Persistent High-Risk Pattern) vs. Class 1 (Rapid Improvement-Stable Pattern): Older age, polypharmacy, and a greater number of missing teeth were independently associated with membership in Class 3. Each additional year of age increased the odds of being in Class 3 by 12% (aOR = 1.12, 95% CI: 1.04–1.21); each additional medication increased the odds by 25% (aOR = 1.25, 95% CI: 1.05–1.49); and each additional missing tooth was associated with a 9% increase in the odds of being classified into Class 3 (aOR = 1.09, 95% CI: 1.02–1.16, *p* = 0.011). Details are presented in Table 8.

**Table 8:** Multivariable logistic regression analysis of determinants for oral frailty trajectory membership.

Variable	Category/comparison	Class 2 vs Class 1		Class 3 vs Class 1	
		aOR (95% CI)	*p*-value	aOR (95% CI)	*p*-value
Age	Per 1-year increase	1.03 (0.96–1.10)	0.408	1.12 (1.04–1.21)	0.003
Number of medications	Per 1-drug increase	1.10 (0.93–1.30)	0.265	1.25 (1.05–1.49)	0.012
Number of missing teeth	Per 1-tooth increase	1.03 (0.98–1.09)	0.240	1.09 (1.02–1.16)	0.011

aOR, Adjusted odds ratio; CI, confidence interval.

### 3.7 Sensitivity analysis

#### 3.7.1 Bootstrap internal validation of multivariable logistic regression

To assess the robustness of the results from the multivariable logistic regression analysis, an internal validation of the model was performed using the Bootstrap method (1,000 re-samples). This method evaluates the stability of parameter estimates and the accuracy of confidence intervals through repeated sampling with replacement. The results of the Bootstrap internal validation are presented in Table 9. The data show that the Bootstrap-adjusted odds ratios (aORs) for all variables were highly consistent with the estimates from the original model, with relative biases all less than 2%. Specifically, the effect sizes for age, number of medications, and number of remaining teeth in the Class 3 vs. Class 1 comparison remained stable across the repeated samples. Furthermore, the 95% confidence intervals generated by the Bootstrap percentile method and the bias-corrected and accelerated (BCa) method were essentially consistent with those from the traditional Wald method, indicating good convergence and stability of the model estimates.

**Table 9:** Bootstrap internal validation results.

Variable	Comparison	Original aOR (95% CI)	Bootstrap aOR (95% CI)	Stability
Age (years)	Class 2 vs. Class 1	1.03 (0.96–1.10)	1.03 (0.95–1.11)	Stable
	Class 3 vs. Class 1	1.12 (1.04–1.21)	1.12 (1.03–1.20)	Stable
Number of medications (types)	Class 2 vs. Class 1	1.10 (0.93–1.30)	1.10 (0.92–1.31)	Stable
	Class 3 vs. Class 1	1.25 (1.05–1.49)	1.25 (1.04–1.48)	Stable
Number of missing teeth (per 1-tooth increase)	Class 2 vs. Class 1	1.03 (0.98–1.09)	1.03 (0.97–1.10)	Stable
	Class 3 vs. Class 1	1.09 (1.02–1.16)	1.09 (1.02–1.17)	Stable

#### 3.7.2 Sensitivity analysis of the latent class growth model

To further validate the robustness of the LCGM trajectory classification, the following sensitivity analyses were conducted. First, OFI-8 scores were treated as count variables and re-fitted using a Poisson-distributed latent class growth model with robust standard errors to account for potential overdispersion. The results showed that the Poisson model also identified three trajectory classes, and the morphological characteristics and class probabilities were highly consistent with the primary analysis (kappa coefficient = 0.92). Second, OFI-8 scores were categorized into tertiles as ordinal variables (low: 0–3; medium: 4–6; high: 7–9) and re-fitted using a multinomial-distributed growth model. The results demonstrated that the trajectory classification was largely consistent with the primary analysis (kappa coefficient = 0.89). Together, these sensitivity analyses support the robustness of the main findings of this study.

## 4 Discussion

Through a 12-month follow-up of 211 older patients with implant-supported prostheses, this study employed latent class growth modeling to identify three distinct trajectory patterns of oral frailty development. This finding suggests that the functional recovery pathways following prosthetic rehabilitation exhibit substantial heterogeneity among patients, rather than conforming to a single “average improvement” pattern. The majority of patients (Class 1, approximately 42.7%) demonstrated an ideal “rapid improvement-stable” trajectory, characterized by a sharp decline in oral frailty scores after prosthesis delivery followed by sustained low levels, suggesting that oral function may have achieved notable improvement after implant rehabilitation. However, it is noteworthy that more than half of the patients (Classes 2 and 3) did not reach this ideal endpoint. The trajectory of Class 2 (gradual improvement-plateau, approximately 35.1%) indicated that the improvement process in a subset of patients was slow and tended to stagnate in later stages, suggesting the presence of unidentified hindering factors. Of greatest clinical concern was Class 3 (persistent high-risk, approximately 22.2%), in which oral frailty levels remained elevated throughout the entire observation period, suggesting that tooth replacement alone may be insufficient to reverse the declining oral function in these patients. Subsequent multivariable analysis further revealed that age, number of medications, and number of missing teeth were independently associated with distinguishing these trajectories, particularly with membership in Class 3. Taken together, these results depict a more complex picture: the oral functional outcomes of older patients are likely shaped by multiple factors, including prosthetic treatment, systemic health status, and oral baseline conditions.

Regarding the causes of the heterogeneous trajectories, the data from this study can only provide evidence of associations between variables and cannot confirm the underlying causal mechanisms. The following discussion of potential mechanisms is based on inferences from the existing literature rather than direct findings from this study. The association between age and adverse trajectories is clinically plausible. It has been reported in the literature that aging may be accompanied by physiological changes such as diminished tissue repair capacity, declined neuromuscular coordination, and systemic inflammaging (18–20). Theoretically, these changes may impair the ability of older patients to adapt to new prostheses and maintain oral homeostasis. However, this study did not measure these biological indicators, and therefore cannot confirm them as mediating pathways. As an indicator of comorbidity burden, the association between number of medications and adverse trajectories may involve multiple potential pathways. It has been reported that polypharmacy, particularly medications with anticholinergic effects, is associated with the development of xerostomia (21, 22). Dry mouth may affect the comfort and efficiency of chewing and swallowing, and may disrupt the oral microbiome, increasing the risk of caries and periodontal disease in remaining teeth (23, 24). However, this study did not measure salivary flow rate, xerostomia severity, or oral microbiome composition, and therefore cannot confirm whether these mechanisms serve as mediating pathways through which medication use influences oral frailty trajectories. The independent predictive effect of medication count on Class 3 membership found in this study suggests that incorporating pharmaceutical assessment into comprehensive oral treatment planning may have clinical value, but this requires future interventional studies to verify. The number of missing teeth intuitively reflects the patient’s existing oral reserve capacity. A greater number of missing teeth may imply a heavier masticatory burden to be borne by the prosthesis, and may also be associated with the patient’s long-term oral health maintenance behaviors or historical dental disease burden (25, 26). However, this study did not systematically collect patients’ previous oral hygiene habits or dental visit history, and therefore cannot differentiate the independent contributions of missing teeth from these behavioral factors. These three factors—age, polypharmacy, and missing teeth—do not exist in isolation, and complex interactions and synergistic effects may exist among them (27, 28). For example, advanced age is often accompanied by more chronic diseases and polypharmacy; xerostomia induced by polypharmacy may accelerate the loss of remaining teeth; and tooth loss may affect nutritional status, which in turn may adversely affect systemic disease control. It should be emphasized that the above discussion of interactive effects is speculative and based on existing literature. The multivariable model in this study only suggests that each of these factors independently contributes to the formation of adverse trajectories, but their interactions were not directly tested. Therefore, the “persistent high-risk” state represented by Class 3 can be understood as patients being embedded in a vulnerability network composed of “systemic aging and comorbidity,” “medication side effects,” and “poor oral baseline conditions,” within which local prosthetic intervention alone may have limited effectiveness. Although this interpretation is clinically intuitive, it requires further research to validate.

Placing this study within a broader academic context, its findings both echo and extend existing research. In recent years, studies on geriatric syndromes, including frailty and sarcopenia, have increasingly emphasized their dynamic evolution and heterogeneous trajectories. This study successfully applied this analytical paradigm to the specific domain of “oral frailty,” focusing on the particular clinical context of “post-implant rehabilitation,” thereby offering a new perspective for understanding the dynamic changes in oral health. Previous cross-sectional studies have reported associations between age, medication count, and oral dryness as well as oral health-related quality of life (22, 29). The longitudinal design of this study advances these associations one step further, suggesting that these factors may serve as predictors of long-term functional trajectory patterns, providing a basis for early identification of high-risk populations. When compared with the few existing studies on oral health trajectories, the intermediate category identified in this study—“gradual improvement-plateau” (Class 2) holds particular significance. These patients neither recovered rapidly nor continued to deteriorate, but rather maintained a “suboptimal” stable functional state. This may represent a potential “window of opportunity” for clinical intervention; however, interventional studies are needed to determine whether investigating the underlying causes and providing enhanced support for such patients can prevent further functional decline. Furthermore, by incorporating both systemic risk factors and oral baseline conditions into a unified analytical framework through multivariable regression, and by validating the model’s robustness using the bootstrap method, this study enhances the methodological reliability of its findings. This approach also resonates with the holistic medical concept that oral health is a window to systemic health.

Based on the above analysis and discussion, the findings of this study offer certain implications for clinical practice and future research directions. First, the associations of age, number of medications, and number of missing teeth with adverse trajectories suggest that clinicians should pay greater attention to older patients with polypharmacy and extensive tooth loss prior to implant treatment, as these factors may help identify individuals requiring prioritized follow-up. Second, the association between medication count and adverse trajectories suggests that medication management may represent a potential entry point for improving oral functional outcomes; however, this requires verification through future interventional trials. The heterogeneity of trajectories indicates that patients’ postoperative recovery pathways differ, and a “one-size-fits-all”follow-up strategy should be avoided in clinical practice. Nevertheless, it must be emphasized that this study is observational in design, and the above recommendations are based on associations revealed by the data rather than strategies validated through interventional trials. Future randomized controlled trials or quasi-experimental studies are needed to examine whether intensive management programs for high-risk populations—such as multidisciplinary collaboration, medication review, personalized maintenance, nutritional counseling, and functional exercise—can effectively improve their oral frailty trajectories.

Of course, this study has several limitations that point to directions for future research. First, this study is an observational longitudinal study without a non-implant control group (e.g., patients receiving conventional removable dentures or no restoration), and the baseline was set on the day of prosthesis delivery rather than before implant treatment. Therefore, the observed changes may partly reflect adaptation to the prosthesis, regression to the mean, or the effects of concurrent follow-up care, and cannot be directly attributed to the implant rehabilitation itself. Furthermore, due to the lack of pre-treatment OFI-8 measurements, it is not possible to assess changes in oral frailty over the complete period from before treatment to after prosthesis delivery, nor to exclude the influence of preoperative procedures (e.g., tooth extraction, temporary restorations, surgical trauma recovery) on baseline measurements. Second, the sample size of this study (n = 211) is relatively limited for latent class growth modeling, particularly with the persistent high-risk group containing only 47 patients (22.2%), which may affect the precision of trajectory parameter estimates and the statistical power of subgroup analyses. Moreover, although the 12-month observation period captures the major post-rehabilitation trends, longer-term follow-up would help reveal the long-term stability of the trajectories, especially whether new turning points emerge after the “plateau” phase. Third, this study employed a complete-case analysis, including only patients who completed all five follow-up visits. Although the overall attrition rate (9.1%) is within an acceptable range and no significant differences in baseline characteristics were observed between completers and dropouts, the dropout sample was small (*n* = 21), limiting the statistical power of between-group comparisons and precluding the complete exclusion of attrition bias on trajectory estimates. Fourth, the “classify-analyze” two-step approach used in this study does not account for classification error. Although classification quality was high (entropy = 0.892, average posterior probabilities >0.9), future studies are advised to adopt a three-step approach to reduce bias. Additionally, this study did not establish an external validation cohort, and the generalizability of the trajectory classification to other populations remains unverified. Fifth, the OFI-8 was originally developed in community-dwelling older adults, and its psychometric properties in patients with implant-supported fixed prostheses require further validation. In this study, the item regarding denture use was removed to tailor the scale to the implant population, and the longitudinal measurement invariance of the adapted scale was formally evaluated. The results supported weak and strong invariance, whereas strict invariance was not supported, suggesting that the construct meaning and item thresholds of the OFI-8 (7-item version) were generally consistent across follow-up time points and that cross-time comparisons were valid. Nevertheless, evidence for strict invariance remains insufficient, and future studies with larger samples or in other older adult populations are warranted to further validate the psychometric properties of this scale. Sixth, the multivariable model in this study only considered the main effects of each predictor and did not test for interactions between variables (e.g., age × number of medications, number of missing teeth × nutritional status). Theoretically, such interaction effects may provide additional explanatory power for trajectory membership; for example, older patients with polypharmacy may face an even higher risk of persistent high-risk trajectory. Future studies with larger sample sizes could explore the inclusion of interaction terms or employ machine learning methods (e.g., random forest, LASSO regression) to screen for important predictors, thereby more comprehensively revealing factors influencing trajectories. Seventh, the study participants were older implant patients who were able to complete the 12-month follow-up, and thus may be relatively healthier and more compliant. Consequently, the generalizability of the findings may be limited and difficult to extend to more frail, multimorbid, or less adherent older populations. Future research could expand sample sizes through multicenter designs, integrate more comprehensive geriatric assessment tools, adopt a three-step approach to handle classification error, and explore associations between biomarkers such as salivary flow rate and the oral microbiome with functional trajectories, thereby gaining deeper insights into the underlying biological mechanisms. Although several key variables were included, some potentially important factors remain unmeasured, such as patients’ socioeconomic status, cognitive function, fine motor skills, social support networks, and specific medication types and anticholinergic burden. These factors may indirectly shape oral functional trajectories by influencing patients’ self-care behaviors, treatment adherence, and physiological responses.

## 5 Conclusion

This study identified heterogeneous trajectories of oral frailty development in older patients with implant-supported prostheses, revealing three distinct patterns: rapid improvement-stable, gradual improvement-plateau, and persistent high-risk. Increased age, greater number of medications, and a higher number of missing teeth were independently associated with membership in the persistent high-risk trajectory. These findings suggest that oral functional recovery following implant rehabilitation is not a homogeneous process, and that patients’ systemic health status and oral baseline conditions are important factors shaping their recovery trajectories. Clinicians should be attentive to these risk factors and strengthen postoperative follow-up and support for high-risk patients. Future research should further explore individualized intervention strategies tailored to different trajectory types and evaluate their effectiveness.

## Data Availability

The original contributions presented in the study are included in the article/supplementary material, further inquiries can be directed to the corresponding authors.
